# Effect of Casting Process and Thermal Exposure on Microstructure and Mechanical Properties of Al-Si-Cu-Ni Alloy

**DOI:** 10.3390/ma17184598

**Published:** 2024-09-19

**Authors:** Peijie Xiao, Shiwei Xu, Longbao Chen, Yu Liu, Jianyu Li, Zhi Xiao, Xianming Meng

**Affiliations:** 1 State Key Laboratory of Advanced Design and Manufacturing Technology for Vehicle, College of Mechanical and Vehicle Engineering, Hunan University, Changsha 410082, China; xiaopeijie@hnu.edu.cn (P.X.); xushiwei@hnu.edu.cn (S.X.); chenlb2022@hnu.edu.cn (L.C.); liuyumail@hnu.edu.cn (Y.L.); hnuxiao@163.com (Z.X.); 2 Suzhou Research Institute of Hunan University, Suzhou 215131, China; 3 China Automotive Technology & Research Center Co., Ltd., Tianjin 300000, China

**Keywords:** heat-resistant aluminum alloy, squeeze casting, thermal exposure, microstructure, mechanical properties

## Abstract

This paper employed squeeze-casting (SC) technology to develop a novel Al-7Si-1.5Cu-1.2Ni-0.4Mg-0.3Mn-0.15Ti heat-resistant alloy, addressing the issue of low room/high temperature elongation in traditional gravity casting (GC). Initially, the effects of SC and GC processes on the microstructure and properties of the alloy were investigated, followed by an examination of the evolution of the microstructure and properties of the SC samples over thermal exposure time. The results indicate that the SC process significantly improves the alloy’s microstructure. Compared to the GC alloy, the secondary dendrite arm spacing of the as-cast SC alloy is refined from 50.5 μm to 18.5 μm. Meanwhile, the size and roundness of the eutectic Si phase in the T6-treated SC alloy are optimized from 11.7 μm and 0.75 μm to 9.5 μm and 0.85 μm, respectively, and casting defects such as porosity are reduced. Consequently, the ultimate tensile strengths (UTSs) at room temperature and at 250 °C of the SC alloy are 5% and 4.9% higher than that of GC alloy, respectively, and its elongation at both temperatures shows significant improvement. After thermal exposure at 250 °C for 120 h, the morphology of the residual second phase at the grain boundaries in the SC alloy becomes more rounded, but the eutectic Si and nano-precipitates undergo significant coarsening, resulting in a 49% decrease in UTS.

## 1. Introduction

As engine pistons, cylinder blocks, and other automotive components operate for extended periods under high temperatures, the harsh working environment imposes higher demands on the heat resistance, wear resistance, and high-temperature dimensional stability of the materials used [1,2,3]. However, the performance of commonly used cast heat-resistant aluminum alloys such as ZL108, A319, A383, and A390, both domestically and internationally, is not sufficiently outstanding. Generally, their high-temperature strength is below 200 MPa at 250 °C and below 150 MPa at 300 °C, which can no longer meet the increasingly stringent service performance requirements [4,5,6,7,8]. Therefore, the research and development of new high-performance, heat-resistant aluminum alloys are of great significance for the advancement of the transportation industry.

Al-Si-Cu-Ni alloys have the advantages of high strength, low density, good heat resistance, and a low coefficient of thermal expansion, making them highly promising for applications in heat-resistant components of transportation vehicles [9,10,11,12]. The authors previously developed a new high-strength, heat-resistant Al-7Si-1.5Cu-*x*Ni-0.4Mg-0.3Mn-0.15Ti (*x* = 0~0.9 wt.%) alloy and found that the Ni content significantly affected the microstructure and properties of the Al-Si-Cu-Ni alloy prepared by gravity casting (GC) [11]. The results showed that Ni can form thermally stable, Ni-rich phases such as Al_3_Ni, Al_3_CuNi, and Al_7_Cu_4_Ni within the GC alloy. A higher Ni content can enhance the high-temperature strength of the GC alloy but will significantly reduce its ductility, thereby weakening its reliability in use. Zuo et al. also found that the Ni content (>1 wt.%) can further improve the room/high temperature strength of Al-12Si-0.9Cu-*x*Ni-0.8Mg, but unfortunately its room temperature elongation is less than 1%, thus limiting its application [12].

Squeeze-casting technology, an important metal processing method, has been widely used in modern industrial production [13,14]. It has significant advantages in improving the performance of aluminum alloy materials, refining the microstructure and enhancing the manufacturing efficiency. Many studies have shown that the squeeze-casting process can refine the α-Al grains and second phases and reduce casting defects, thereby improving the ductility of the alloy while further enhancing its strength [9,15,16,17]. Therefore, applying squeeze-casting technology to the preparation of Al-Si-Cu-Ni alloy holds the potential to achieve high strength while maintaining high ductility. In particular, the application of squeeze-casting technology is expected to improve the microstructure of the alloy, thereby enhancing its high-temperature mechanical properties and heat resistance.

In this paper, a newly self-developed, heat-resistant Al-7Si-1.5Cu-1.2Ni-0.4Mg-0.3Mn-0.15Ti (referred to as Al-Si-Cu-Ni) alloy is taken as the object of study, and the influence of two forming processes (i.e., squeeze casting and gravity casting) on the microstructure and properties of the alloy is investigated. Subsequently, thermal exposure tests are conducted on the squeeze-cast samples to study the evolution of their microstructures and properties with exposure time.

## 2. Experimental Procedure

**Figure 1:**
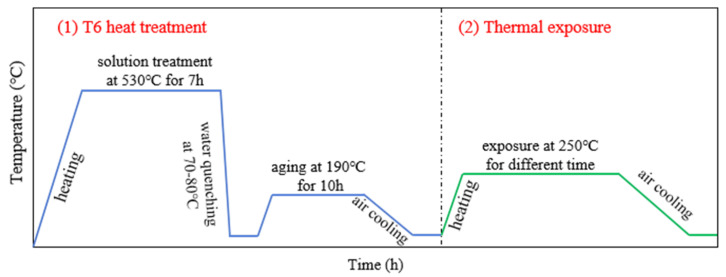
A schematic diagram illustrating the T6 heat treatment and thermal exposure process curves for the Al-Si-Cu-Ni alloy.

Based on previous research [11,18], the Al-Si-Cu-Ni ingots were prepared by gravity casting and squeeze casting, with applied pressures of 0 MPa and 100 MPa, respectively. Then, these ingots, with diameters of Φ60 mm, were strengthened by suitable T6 heat treatment (i.e., solution treatment at 530 °C for 7 h, followed by water quenching at 70–80 °C, and finally aging at 190 °C for 10 h) using a KSL-1200X Box Furnace (HE FEI KE JING MATERIALS TECHNOLOGY CO., Ltd., Hefei, China). Subsequently, multiple squeeze-cast samples that had undergone T6 heat treatment were placed in a preheated furnace at 250 °C and quickly removed at different intervals to obtain samples with varying thermal exposure times, as shown in Figure 1. Considering that the longer the thermal exposure time, the closer the microstructure approaches equilibrium and the slower the rate of change, increasing time intervals were selected in this study, specifically 0 h, 2 h, 5 h, 10 h, 20 h, 40 h, 70 h, and 120 h.

Small samples were collected from the same center location of each casting, ground and polished using varying grits of sandpaper and diamond polishing paste, and etched with a 0.5% HF aqueous solution. Subsequently, these samples were examined for metallographic and SEM (Scanning Electron Microscope) microstructures using a Cai Kang DMM-490C optical microscope (Shanghai, China) and a Gemini SEM300 field emission scanning electron microscope (Carl Zeiss AG, Oberkochen, Germany). The metallographic images were analyzed and quantified using Image-Pro Plus 6.0 software, and at least six metallographic images from different locations of each specimen were captured for the measurement of average grain size and secondary dendrite arm spacing. The phase composition of the alloy was analyzed using a SHIMADZU XRD-7000S diffractometer (SHIMADZU, Kyoto, Japan), with a scanning angle range of 20–90° and a scanning rate of 5°/min. A Tecnai G2F30 Transmission electron microscope (TEM, FEI, Hillsboro, OR, USA) was used to observe nano-precipitates in the specimens before and after thermal exposure. Room temperature tensile tests were conducted with an MTS C45.105EY testing machine equipped with a Haitham optical extensometer (measurement accuracy: 1 μm), at a tensile speed of 1 mm/min. The room temperature tensile test method and specimen dimensions are in accordance with the Chinese standard GB/T228.1-2010 (equivalent to ASTM A370-2016) [9], while three tensile samples were selected for mechanical properties testing to obtain the average value, ensuring the accuracy of the experiment.

## 3. Results and Discussion

### 3.1. Effect of Forming Process on the Microstructure of Al-Si-Cu-Ni Alloy

**Figure 2:**
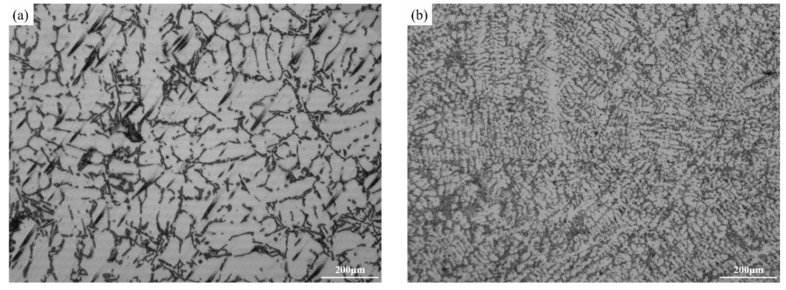

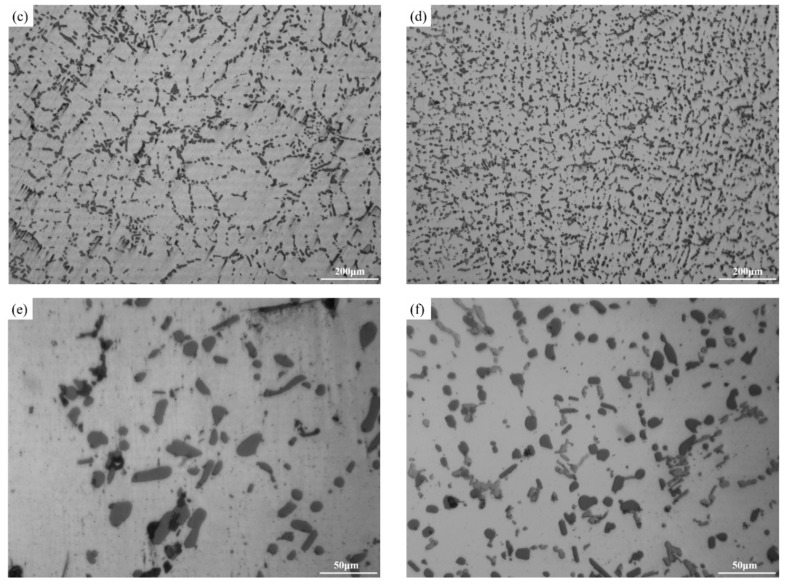
Optical microstructure of the GC and SC alloys in as-cast and T6-treated states: (**a**) GC-As-cast, (**b**) SC-As-cast, (**c**,**e**) GC-T6, and (**d**,**f**) SC-T6.

**Figure 3:**
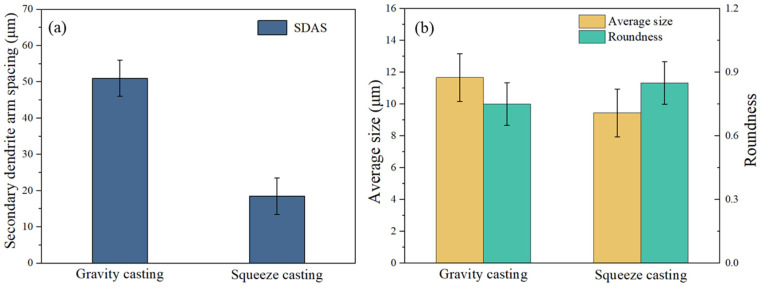
Quantitative statistics of the microstructure of the GC and SC alloys: (**a**) Secondary Dendrite Arm Spacing (SDAS) in as-cast alloys, (**b**) Size and roundness of eutectic Si in T6-treated alloys.

Figure 2 shows the optical microstructure of the as-cast and T6-treated Al-Si-Cu-Ni alloys prepared by gravity casting (GC) and squeeze-casting (SC) processes. As shown in Figure 2a, coarse α-Al dendrites and plate-like or needle-like eutectic Si dominate in the as-cast GC alloy, with some composition segregation. After T6 heat treatment, the plate-like and needle-like eutectic Si in the GC alloy undergoes melting and significant refinement and spheroidization (Figure 2c). Compared to the as-cast GC alloy, the grain size of the as-cast SC alloy is significantly reduced. Quantitative statistics show that the average secondary dendrite arm spacing (SDAS) of the α-Al grains decreases from 50 μm to 18.5 μm, with a reduction of 63% (Figure 2b and Figure 3a). Compared to the T6-treated GC alloy, the eutectic Si in the T6-treated SC alloy becomes smaller and more rounded. The quantitative statistics show that the average size and the roundness of the eutectic Si improve from 11.7 μm and 0.75 μm to 9.5 μm and 0.85 μm, respectively. Additionally, there is less composition segregation and a more uniform distribution of the microstructure in the T6-treated SC alloy (Figure 2c–f and Figure 3b).

**Figure 4:**
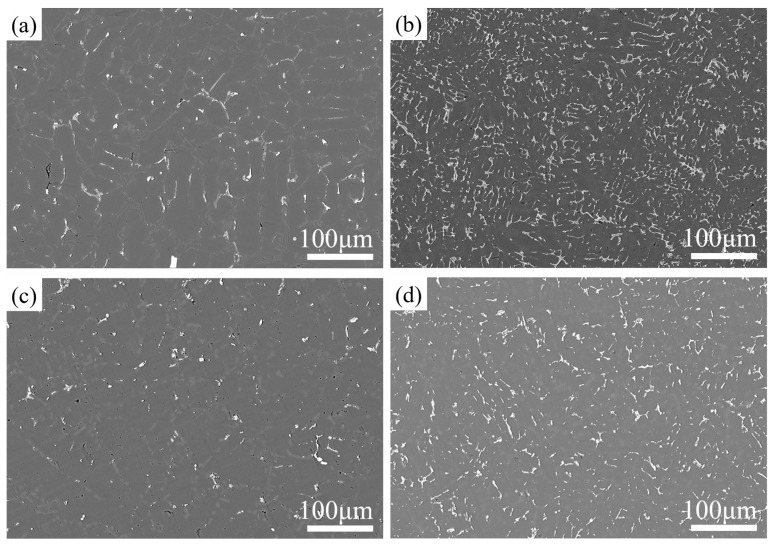
SEM images of the GC and SC alloys in the as-cast and T6-treated states: (**a**) GC-As-cast, (**b**) SC-As-cast, (**c**) GC-T6, and (**d**) SC-T6.

**Figure 5:**
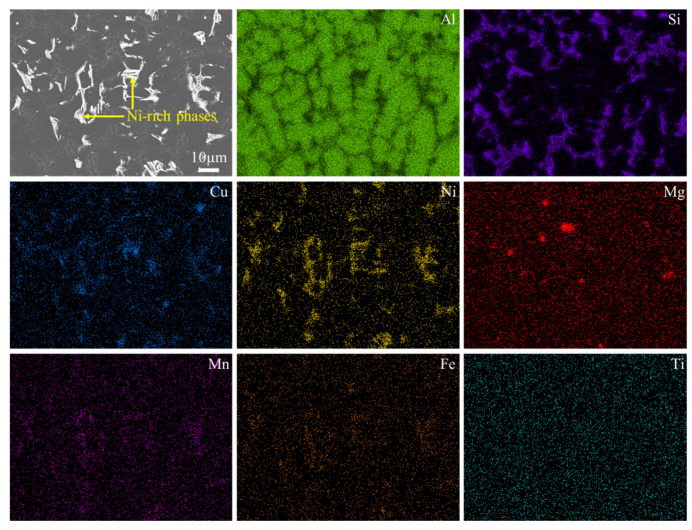
Elemental surface distribution map of the high-magnification region of the as-cast SC alloy.

Figure 4 shows the SEM images of the as-cast and T6-treated Al-Si-Cu-Ni alloys prepared by two casting processes. As shown in Figure 4a,c, the microstructure of the as-cast GC alloy mainly consists of a dark α-Al matrix, a eutectic Si phase, and a bright second phase, with a small number of pores also observable in the GC alloy. After squeeze casting (SC), the distribution of the bright second phase in the as-cast SC alloy becomes more uniform, and its volume fraction increases. This may be attributed to the reduced solubility of the Cu atoms in α-Al under pressure or to a shift in the eutectic point position of the alloy phase diagram due to the applied pressure [19,20]. Zhang et al. also found that the volume fraction of the second phase in the squeeze-cast alloy continuously increased with increasing applied pressure [20]. According to the elemental surface distribution in the high-magnification region (Figure 4), these bright second phases mainly consist of Ni-rich phases such as ε-Al_3_Ni, Al_3_CuNi, and a small amount of AlSiMnFe phases. Meanwhile, the Ti element is uniformly distributed without significant enrichment, indicating that it is mainly dissolved in α-Al matrix, which may play a role in grain refinement. From the comparative analysis in Figure 5, it can be also seen that there exists a small amount of Mg elemental enrichment, which overlaps with the distribution of Si and Cu elements. Therefore, it can be hypothesized that trace amounts of the Q (AlCuMgSi) phase may be generated in the alloy after heat treatment, which is consistent with the results of previous studies [21,22]. In addition, there are almost no pores in the SC alloy, which is because the SC pressure can promote compensatory shrinkage during the solidification process of the alloy, thereby reducing or even eliminating micropores. After the T6 treatment, the size and the number density of the second phase decrease significantly. This may be due to the decomposition of some second phases during the solution treatment process at high temperatures, where some alloying elements dissolve into the α-Al matrix and form a supersaturated solid solution after quenching. The strengthening phases precipitated during the aging process are at the submicron or even nanometer level, which are difficult to observe under scanning electron microscopy.

**Figure 6:**
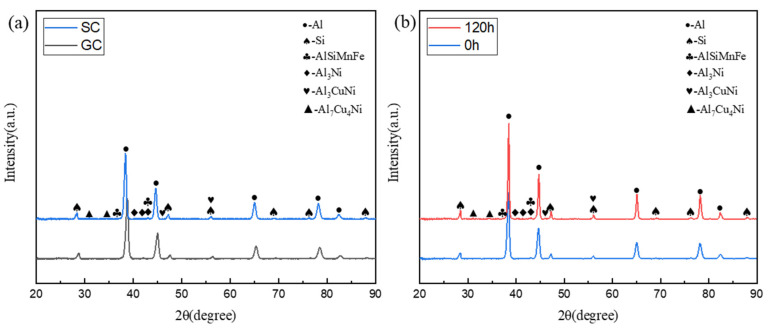
XRD patterns of the alloys prepared by different processes: (**a**) as-cast state and (**b**) T6-treated state.

Figure 6a shows the XRD patterns of the as-cast Al-Si-Cu-Ni alloys prepared by two different casting processes. Obviously, the microstructure of the Al-Si-Cu-Ni alloy is mainly composed of α-Al, eutectic Si, AlSiMnFe, and Ni-rich phases. The comparative analysis of the XRD patterns of the GC and SC alloys shows that the angle of the diffraction peaks of the α-Al phase is slightly shifted to the left. This may be attributed to the decrease in the solubility of Cu in the SC alloy, leading to an increased interplanar spacing of the α-Al crystal lattice. Studies have shown that the incorporation of Cu atoms into α-Al can result in a decrease in the interplanar spacing, and thus, the smaller the number of Cu atoms dissolved into α-Al, the larger the crystal plane spacing [23]. This is also in better agreement with the microstructural changes described in Figure 4. Apart from this, there are no significant changes in the intensities and angles of the derived peaks of other constituent phases, indicating that the casting process does not significantly alter the phase composition of the alloy. Figure 6b shows the XRD patterns of the T6-treated SC alloy after thermal exposure for 0 h and 120 h. The microstructure of the T6-treated alloy is primarily composed of α-Al, eutectic Si, AlSiMnFe, and Ni-rich phases. By comparing the patterns of the alloy before and after 120 h of thermal exposure, it can be observed that there are no significant changes in the intensities and angles of the derived peaks of these phases. This indicates that the phase composition of the alloy remains unchanged before and after thermal exposure.

### 3.2. Effect of Forming Process on Mechanical Properties of Al-Si-Cu-Ni Alloy

**Figure 7:**
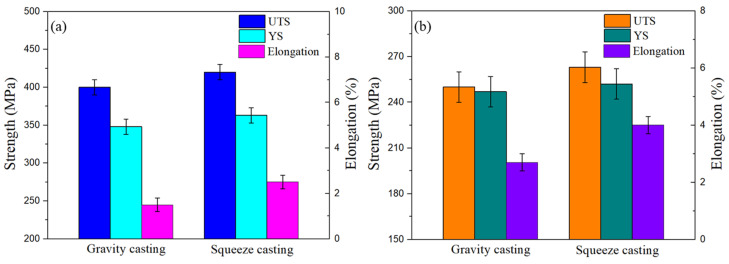
Mechanical properties of the alloys prepared by different casting processes: (**a**) mechanical properties at room temperature and (**b**) mechanical properties at 250 °C.

Figure 7 shows the room temperature and high-temperature (250 °C) mechanical properties of the T6-treated Al-Si-Cu-Ni alloy prepared by two casting processes. The room-temperature ultimate tensile strength (UTS), the yield strength (YS), and the elongation (El) of the GC alloy were 400 MPa, 348 MPa, and 1.5%, respectively. After squeeze casting, the room temperature UTS, YS, and El of the alloy increased to 420 MPa, 363 MPa, and 2.3%, respectively, which were 5.1%, 4.2%, and 53.3% higher than those of the GC alloy. The high-temperature (250 °C) UTS, YS, and El of the GC alloy were 250 MPa, 247 MPa, and 2.7%, respectively, while those of the SC alloy were 263 MPa, 252 MPa, and 4.0%, respectively. Compared to the GC alloy, the high-temperature properties of the SC alloy were increased by 4.9%, 2.0%, and 48.1%, respectively.

Compared to traditional gravity casting, the SC alloy exhibits superior strength and elongation at both room temperature and high temperature (250 °C). This is mainly attributed to the reduction in internal shrinkage and porosity and the refinement of α-Al grains and eutectic Si, as well as a more uniform distribution of the second phase in the SC alloy. In the SC alloy, the liquid melt solidifies under applied pressure, which enhances the melt’s feeding ability and densifies the microstructure (e.g., significant reduction in microporosity, shrinkage, and gas porosity), thereby enhancing the alloy’s mechanical properties. Additionally, the closer contact between the molten metal and the mold under applied pressure increases the effective contact area and interface heat-transfer coefficient so that the cooling rate of the melt increases, resulting in grain refinement [24,25]. Moreover, the feeding and the dynamic solidification of the melt under pressure induce relative flow, which accelerates the detachment, fragmentation, and proliferation of grains, significantly increasing the nucleation rate and refining the grains [26]. Generally, grain boundaries hinder the movement of dislocations and the propagation of microcracks. Finer grains have larger grain boundary curvature and total area, resulting in stronger hindrance to dislocation motion and microcrack propagation. This contribution follows the Hall–Petch empirical formula [27,28]:

(1) $$\sigma_{s}=\sigma_{0}+Kd^{-1/2}$$

In the formula, *d* represents the average grain diameter, *K* is a constant that characterizes the effect of the grain boundaries on strength and is related to the grain boundary structure, *σ*_0_ represents the resistance to deformation within the grains, and *σ_s_* is the yield strength of the alloy.

It can be seen from the above formula that the smaller the grain size of the alloy, the higher the yield strength of the alloy. At the same time, due to the finer grains, the plastic deformation of the alloy can be dispersed in more grains, making the plastic deformation more uniform to improve the alloy’s plasticity. The refinement of the eutectic Si phase in the SC alloy reduces its splitting effect on the matrix. Additionally, a more uniform distribution of the eutectic Si phase and other secondary phases can also reduce stress concentration when the alloy is subjected to force, thereby improving the strength and plasticity of the alloy. Unfortunately, both GC and SC alloys have room-temperature elongations of less than 4%, which are considered to be typical of brittle fractures. Li et al. showed that an increase in squeeze-casting pressure not only results in finer grains but also in a gradual decrease in porosity, which improves the strength and plasticity of the alloy [13]. In addition to the above effects, Qin et al. found that the squeeze-casting process can also refine the second phases, such as AlSiMnFe and Al_3_CuNi, as well as improve the morphology of the second phases, especially the size of the eutectic Si phase, which can be refined to the submicron or even nanometer scale [9]. They believe that secondary phases generally precipitate at the solid–liquid interface of primary α-Al grains and eventually distribute at the grain boundaries, with their sizes decreasing as the grains refine. Furthermore, due to the high solidification rate of SC alloys, the refinement of primary α-Al grains helps shorten the movement distance of the solid–liquid front, which is beneficial for improving the morphology and distribution of the secondary phases. El-Khair et al. also found that the squeeze-casting process can refine the size of secondary phases and improve their morphology in the Al-Mg-Si alloys [29]. Many studies on the squeeze-casting process of Al-Si alloys have shown that the SC process significantly enhances the room-temperature mechanical properties of the alloy. For example, Chandra et al. reported that the UTS of SC alloys increased by 15% as compared to GC alloys [30], and Pratheesh et al. also claimed that the SC process increased the UTS of Al-Si-Cu-Ni alloys by 33% [31].

The deformation and fracture mechanisms of alloys at high temperatures differ from those at room temperature. The high-temperature elongation of the GC alloy is only 2.7%, which is characteristic of brittle fracture, whereas the SC alloy exhibits a high-temperature elongation of nearly 4%, indicating a mixture of brittle and ductile fracture. As the temperature increases, the energy of the grain boundaries increases while the strength gradually decreases. In addition to dislocation slip, the deformation of the alloy at high temperatures can also involve grain boundary sliding, grain boundary diffusion, and creep. As a result, the strengthening effect brought by grain refinement gradually diminishes until it disappears. Because the contribution of fine-grain strengthening to high-temperature strength is relatively low, the SC process only improves the high-temperature strength at 250 °C by 4.9%. However, at high temperatures, the strengthening effect brought by the reduction in casting defects in the Al-Si-Cu-Ni alloy remains effective, and the heat-resistant phases in the alloy significantly contribute to high-temperature strength. Therefore, as compared to gravity casting, the improvement in the high-temperature mechanical properties of SC alloys is mainly attributed to a denser microstructure and a more uniform distribution of thermally stable secondary phases.

### 3.3. Effects of Heat Exposure on the Microstructure and Properties of the SC Alloy

**Figure 8:**
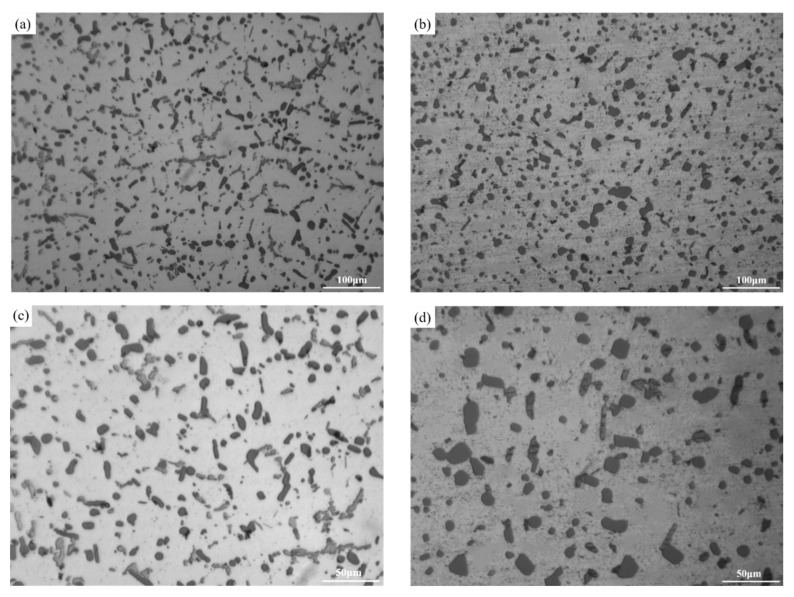
Optical microstructure of the T6-treated SC alloy subjected to thermal exposure at 250 °C for different times: (**a**,**c**) 0 h, and (**b**,**d**) 120 h.

Figure 8 shows the optical microstructure of the Al-Si-Cu-Ni alloy prepared by the squeeze-casting process after 0 h and 120 h of thermal exposure. In Figure 8, the bright areas represent the α-Al matrix, the black granular or spherical phases are the eutectic Si phases, and the gray strip phases are the Ni-rich phases or the AlSiMnFe phases. Before thermal exposure (i.e., in the T6 state), the eutectic Si phase in the alloy appears to be fused from needle-like and flaky forms and spheroidized, and the grain boundary contours are still visible in the low-magnification image (Figure 8a). After thermal exposure for 120 h, the distribution of the eutectic Si phase becomes more uniform, the grain boundary contours are no longer visible, and the morphology becomes more rounded (Figure 8b). Additionally, after thermal exposure for 120 h, some eutectic Si phases show significant coarsening, while some remain small and granular. Based on these observations, it is speculated that all eutectic Si phases may have undergone coarsening during thermal exposure, and the smaller granular eutectic Si observed in Figure 8 is the result of the growth of submicron-sized or nano-sized eutectic Si prepared during the squeeze-casting process. The average equivalent diameters of the eutectic Si phases were quantitatively measured by using Image-Pro Plus 6.0 software. After thermal exposure for 120 h, the average equivalent diameter of the eutectic Si phase was 10.9% higher than that of the alloy without thermal exposure.

**Figure 9:**
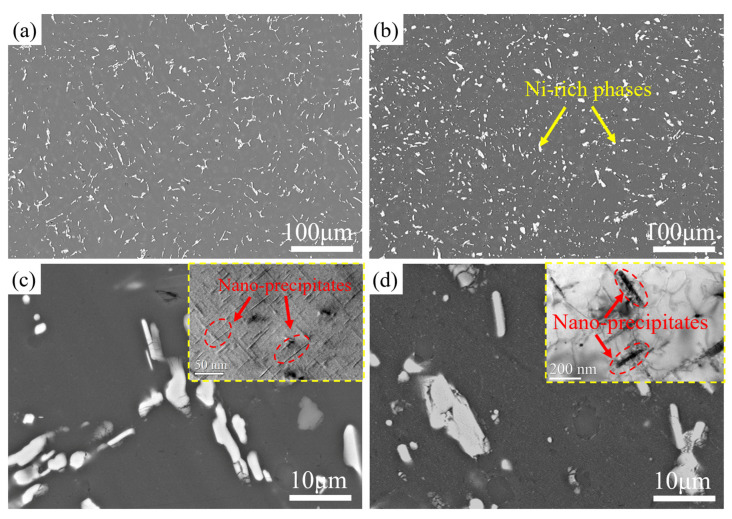
SEM and TEM images of the T6-treated SC alloy subjected to thermal exposure at 250 °C for different times: (**a**,**c**) 0 h and (**b**,**d**) 120 h.

**Figure 10:**
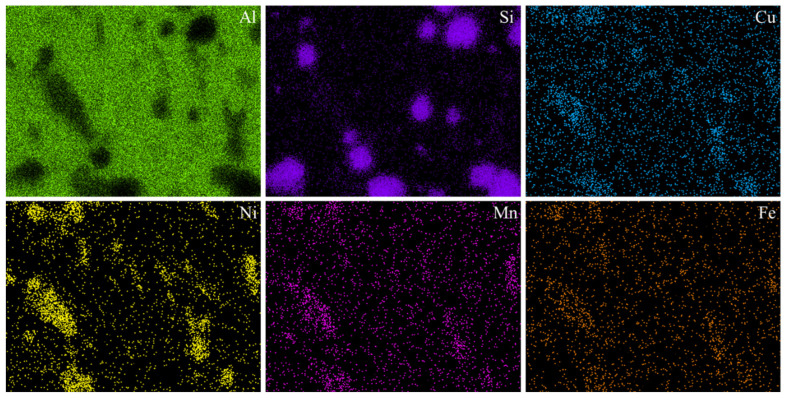
Elemental surface distribution of the T6-treated SC alloy after thermal exposure for 120 h (i.e., the region in Figure 9d).

Figure 9 shows the SEM images of the T6-treated SC alloy subjected to thermal exposure at 250 °C for 0 h and 120 h. Figure 10 exhibits the elemental surface distribution in the region shown in Figure 8d. Obviously, similar to the as-cast alloy, the second phases in the T6-treated alloy before and after thermal exposure are primarily composed of ε-Al_3_Ni and other Ni-rich phases, as well as AlSiMnFe phases. After thermal exposure for 120 h, the morphology of the second phases in the alloy becomes more rounded (i.e., the surface curvature decreases), and the distribution of the second phases in the alloy becomes more uniform. Some second phases coarsen and become blocky, while some remain fine and granular (Figure 9a,b). Regarding the coarsening of the second phases and the rounding of their morphology, it is speculated that this is the result of the second phases changing in the direction of the reducing interface energy during thermal exposure, as a more rounded shape has a lower specific surface area. According to the high-magnification TEM images in the upper right corner of Figure 9c, a large number of nano-sized precipitates with average lengths and thicknesses as small as 25 nm and 2 nm can be seen dispersed in the alloy. However, these precipitates significantly coarsen after thermal exposure for 120 h, with an average length and thickness as large as 150 nm and 10 nm (Figure 9d), respectively, and they are also transformed from a metastable state to a stable state under high energy. Therefore, it is hypothesized that these nano-sized precipitates are θ′/θ-Al_2_Cu phases, which is consistent with previous findings [32,33].

**Figure 11:**
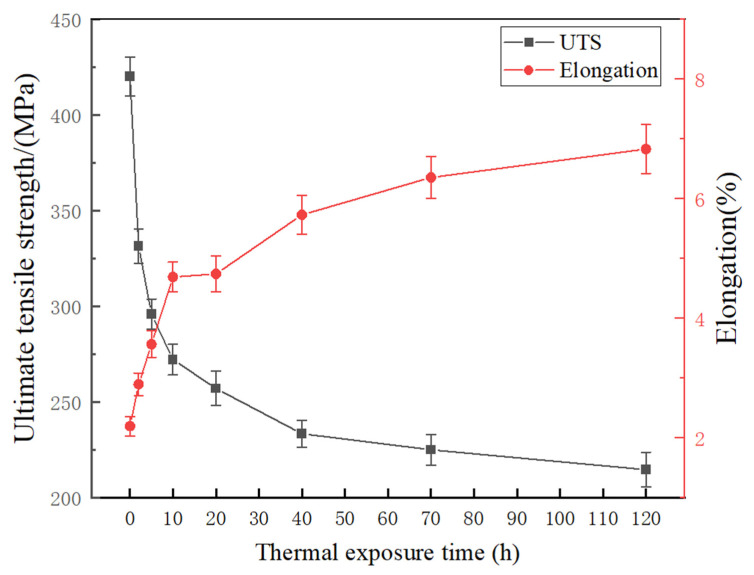
Mechanical properties of the T6-treated SC alloy subjected to thermal exposure at 250 °C for different times.

Figure 11 shows the variation curves of room temperature UTS and the elongation of the T6-treated SC alloy with different thermal exposure times. As the thermal exposure time increases, the UTS of the SC alloy gradually decreases, while the elongation gradually increases. With the thermal exposure time increasing from 0 h to 120 h, the room-temperature UTS of the SC alloy decreases from 420 MPa to 214 MPa, a total decrease of 49%. Within the initial 2 h of thermal exposure, the UTS of the alloy rapidly decreases from 420 MPa to 331 MPa, which is a decrease of 21.2%, accounting for 43.2% of the total decrease. Subsequently, as the thermal exposure time increases from 2 h to 120 h, the strength continues to gradually decrease to 214 MPa and finally tends to a stable value. Compared to the alloy with thermal exposure for 2 h, the UTS of the alloy subjected to thermal exposure for 120 h decreases by 35.3%, accounting for 56.8% of the total decrease. The elongation of the SC alloy increases rapidly during the initial 10 h of thermal exposure, with the elongation increasing from 2.3% to 4.7%. Subsequently, as the thermal exposure time extends from 10 h to 120 h, the elongation increases from 4.7% to 6.8%, indicating that 53.3% of the total increase in elongation occurs within the first 10 h of the thermal exposure period studied.

Two primary reasons explain the significant decline in the mechanical properties of SC alloys after 120 h of thermal exposure. On the one hand, J. da Costa Teixeira et al. demonstrated that the θ′-Al_2_Cu phase is highly resistant to shear, with its primary strengthening mechanism being dislocation bypassing [34]. As the θ′-Al_2_Cu precipitate coarsens during thermal exposure, the critical shear stress for dislocation bypass decreases, reducing resistance and diminishing the precipitation-strengthening effect of the θ′-Al_2_Cu phase. On the other hand, the alloy’s grain boundary strength diminishes in high-temperature environments, leading to grain boundary slip and diffusion. The fine-grain strengthening effect brought by the SC process reduces or even disappears, making the high-temperature stability advantage of the SC alloy less noticeable. Many existing studies also indicate that the high-temperature mechanical properties and the thermal exposure characteristics of the Al-Si series alloys prepared by the squeeze-casting process do not show significant advantages as compared to gravity casting [35,36]. For example, Lin et al. studied the thermal exposure characteristics of squeeze-cast Al-Si-Cu-Mn-Fe alloys and found that, after thermal exposure for 100 h, the strengths of the SC and GC alloys are almost the same. This is due to the reduction of the fine-grain strengthening effect brought on by the SC process, as well as the coarsening and reduction of the number of θ′-Al_2_Cu phases [35].

## 4. Conclusions

To address the challenges in the heat-resistant aluminum alloy industry, this paper proposes increasing the Ni content to 1.2 wt% based on our previously developed alloys and adopting squeeze casting instead of conventional gravity casting. Additionally, the effects of the two casting processes on the microstructure and mechanical properties of the Al-7Si-1.5Cu-1.2Ni-0.4Mg-0.3Mn-0.15Ti alloy, as well as the thermal exposure characteristics of the T6-treated SC alloy, were investigated for the first time. The main conclusions are as follows:

(1) The squeeze-casting process can significantly improve the microstructure of the alloy. Compared to gravity casting, the secondary dendrite arm spacing of the as-cast SC alloy is refined from 50.5 μm to 18.5 μm. Meanwhile, the size and roundness of the eutectic Si phase in the T6-treated SC alloy are optimized from 11.7 μm and 0.75 μm to 9.5 μm and 0.85 μm, respectively. Additionally, squeeze casting can reduce porosity in the alloy, making it denser and ensuring a more uniform distribution of the second phase.
(2) Squeeze casting can enhance the room/high temperature mechanical properties of the alloy. The ultimate tensile strength (UTS) of the T6-treated SC alloy at room temperature and at 250 °C are 420 MPa and 263 MPa, respectively, which is 5% and 4.9% higher than that of GC alloy, and its elongation at both temperatures shows significant improvement.
(3) After thermal exposure at 250 °C for 120 h, the morphology of the residual second phase at the grain boundaries in the SC alloy becomes more rounded, but the eutectic Si and nano-sized precipitates undergo significant coarsening. With the increase of the thermal exposure time from 0 h to 120 h, the UTS of the alloy decreases from 420 MPa to 214 MPa, a total decrease of 49%, while its elongation gradually increases, with the rate of change slowing down and eventually stabilizing.

## Data Availability

The raw/processed data required to reproduce these findings cannot be shared at this time, as the data also form part of an ongoing study.
